# New Ceramics Precursors Containing Si and Ge Atoms—Cubic Germasilsesquioxanes—Synthesis, Thermal Decomposition and Spectroscopic Analysis

**DOI:** 10.3390/molecules27041441

**Published:** 2022-02-21

**Authors:** Aleksandra Skoczeń, Dawid Frąckowiak, Robert E. Przekop, Miłosz Frydrych, Małgorzata Kasperkowiak, Piotr Jeleń, Maciej Sitarz, Bogdan Marciniec

**Affiliations:** 1 Faculty of Chemistry, Adam Mickiewicz University in Poznań, Uniwersytetu Poznańskiego 8, 61-614 Poznań, Poland; skoczen@amu.edu.pl (A.S.); frydrych@amu.edu.pl (M.F.); 2 Centre for Advanced Technologies, Adam Mickiewicz University in Poznań, Uniwersytetu Poznańskiego 10, 61-614 Poznań, Poland; dawidoff@amu.edu.pl (D.F.); or r.przekop@gmail.com (R.E.P.); malgorzata.kasperkowiak@amu.edu.pl (M.K.); 3 Faculty of Materials Science and Ceramics, AGH University of Science and Technology, Al. Mickiewicza 30, 30-059 Kraków, Poland; pjelen@agh.edu.pl (P.J.); msitarz@agh.edu.pl (M.S.)

**Keywords:** silsesquioxanes, germasilsesquioxanes, POSS, thermal analysis, decomposition, sublimation, ceramics precursors, FT-IR, Raman spectroscopy

## Abstract

Compounds of the silsesquioxane type are attractive material precursors. High molecular weights and well-defined structures predestine them to create ceramics with a controlled composition at the molecular level. New molecular precursors of ceramic materials with the ratio of Si:Ge = 7:1 atoms were obtained. The influence of organic substituents on the thermal decomposition processes of germasilsesquioxanes was investigated. Some of the structures obtained are characterized by a high non-volatile residue after the thermal decomposition process. The introduction of the germanium atom to the structure of the silsesquioxane molecular cage reduces the thermal stability of the obtained structures.

## 1. Introduction

**Figure 1:**
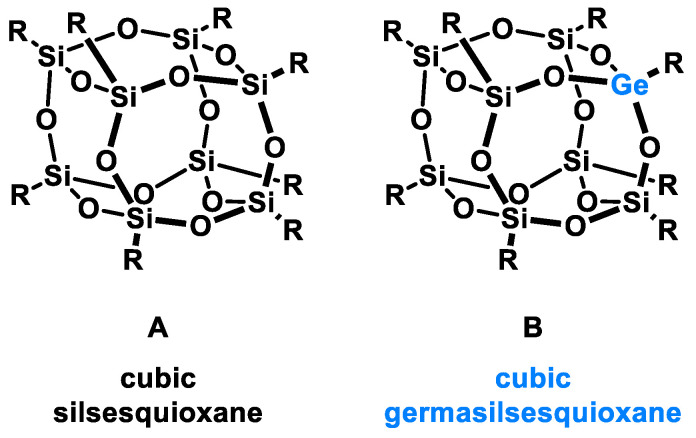
The structures of cubic silsesquioxane (**A**) and germasilsesquioxanes (**B**).

Polyhedral Oligomeric Silsesquioxanes, of the general formula [RSiO_1.5_]_n_, are well-defined, hybrid cage compounds with a three-dimensional structure and nanometric size [1]. Silsesquioxanes have a siloxane core with inert or reactive organic and inorganic functionalities located at silicon atoms. The type of substituents attached to the silsesquioxane skeleton defines many of the physicochemical properties of these systems (e.g., solubility, composite compatibility and thermal and chemical stability) [2,3,4,5,6,7,8]. The most widely described and researched silsesquioxanes classes in literature are silsesquioxanes with a closed structure of the T_8_ type, of which the structure of the silicon core resembles a cube (Figure 1A) [9].

On the other hand, germasilsesquioxanes (Figure 1B) combine the features of silsesquioxanes and the properties of systems containing Si–O–Ge groups, i.e., germasiloxanes [10]. Germasiloxane motifs can be seen as the resultant of siloxane (Si–O–Si) and germoxane (Ge–O–Ge) units in terms of their reactivity and other properties. Germoxanes are analogs of higher molecular weight siloxanes that contain much more reactive linkages than siloxanes; they also show high refractive index values, low dielectric constants, and biocompatibility [11,12]. Compounds with the Si–O–Ge bonds exhibit intermediate properties. The change from the silicon atom to germanium shifts the electron density from the oxygen atom towards silicon, increasing Ge–O bond polarization. Compared to siloxanes, the introduction of the Si–O–Ge group significantly increases the refractive index values, which directly affects the optical properties of the compounds. Such systems can be precursors of the inorganic SiO_2_/GeO_2_ phases used to produce specialized spin glasses, glass films, microlenses, lasers and adhesive layers [13]. From an economic standpoint, germanium-containing compounds will certainly be more expensive than silicon-based compounds. However, it should be emphasized that due to their different properties and possible applications, it is definitely worth examining them carefully.

Many research groups have studied the thermal properties of silsesquioxanes. The influence of the nature of substituents (reactive and inert groups) on the thermal stability and degradation mechanism of various classes of POSS has been investigated. There are reports available on the thermal degradation of silsesquioxanes, e.g., an excellent work of Fina [14]. The thermal parameters have been surveyed for the cubic [14,15,16,17], double-decker [18] and open-caged silsesquioxanes with various functionalities [19]. So far, however, no data have been published on the thermal characteristics of germasilsesquioxanes.

Silsesquioxanes and their derivatives are treated almost as a remedy for all problems related to the properties of materials [1,20] and used as molecular fillers [21,22] or components which improve the physical parameters of given substances [21,22,23,24,25]. However, although they have been studied for many years and by many research groups, they have not found widespread use in material or composite systems. One of a few areas in which they have been effectively applied is in the production of cosmetics [26].

## 2. Results and Discussion

### 2.1. Synthesis of Cubic Germasilsesquioxanes

In our previous studies on germasilsesquioxanes, we focused on obtaining derivatives with reactive functional groups, their spectroscopic characteristics and the functionalization of these compounds. However, there are no reports in the literature concerning the physicochemical properties of germasilsesquioxanes. It has inspired us to synthesize a range of physiochemically well-characterized derivatives of germasilsesquioxanes, which may open new paths for the development of this group of compounds.

**Figure 2:**
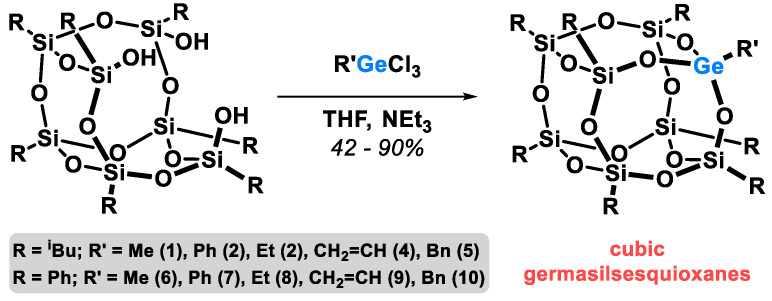
The synthesis of cubic germasilsesquioxanes.

All studied germasilsesquioxanes were synthesized via a corner-capping reaction of cubic silsesquioxane silanetriols with respective organotrichlorogermanes (Figure 2). 

Almost all compounds were obtained in very good and excellent yields (except for octaphenylgermasilsesquioxane **7**, due to its moderate solubility in THF—a trait shared with its silicon analog) and fully characterized spectroscopically (^1^H, ^13^C, ^29^Si NMR and high-resolution mass spectrometry). Compounds **4** and **9** were previously synthesized in our research group and successfully studied in their reactivity toward ruthenium hydride complexes in catalytic *trans*-germylation [27] as well as in olefin cross-metathesis [28]. Compound **3** was only recently obtained by Hreczycho et al. via a new catalytic protocol involving the corner-capping of (2-methylallyl)germanes with silsesquioxane silanols that were catalyzed by Sc(OTf)_3_ [29]. All the obtained germasilsesquioxanes are listed in Table 1.

### 2.2. Thermal Degradation Analysis of Cubic Germasilsesquioxanes

One of the most often discussed physicochemical parameters describing the POSS type compounds is thermal resistance or stability. The majority of authors, however, content themselves with thermogravimetric analysis (usually in the nitrogen atmosphere) and take into account the temperature of the first mass loss, usually of 5% or 1%. This generally repeated approach is due to the fact that POSS compounds were quite widely used as additives to polymeric or composite materials, and the aspects of thermal degradation of silsesquioxane-containing polymer systems were described, taking into account both the thermal transformations of the silsesquioxane component and the polymer matrix itself [30,31,32,33,34,35,36,37]. This approach, however, resulted in adopting a research methodology for the molecular type of materials. However, the silsesquioxane-type compounds are neither composites nor polymers, but most often (except for the so-called mix-cage systems) molecular type compounds. From the point of view of characterizing silsesquioxane as a molecular chemical compound, this approach seems incorrect. The analysis of the phenomena taking place in the temperature gradient should take into account the molecular aspects of the structure and composition of the tested compound. Thus, they should be characterized by such parameters as melting point, boiling point or sublimation point. Because of their relatively large molecular mass and specific structure with a characteristic oxygen-silicon core, the silsesquioxane compounds have slightly different thermal characteristics than typical organic compounds [15]. In normal conditions, both in the oxidizing and inert atmosphere (Ar, N_2_), they do not evaporate, but they often undergo sublimation [17,18,19]. The understanding of the transitions and transformations of silsesquioxane compounds under different thermal and atmospheric (oxidative or non-oxidative) conditions, both physical and chemical, are of utmost importance when considering their application as preceramic materials for special applications, e.g., protective coatings for materials applied in harsh service environments [38,39].

Another important observation is that it is impossible to reliably predict the behavior of a new compound at a temperature gradient for silsesquioxanes. Usually, the thermal behavior of newly obtained compounds complies with the rules established for the substituents, but the thermal processes are not so distinctly defined and unambiguous as those observed for low-molecular organic compounds. The behavior of silsesquioxane compounds can be treated as a hybrid between that of low-molecular organic compounds and polymers. For the silicon structures of the silsesquioxane type, specific numerical models have been devised that—with a high probability—permit prediction of their behavior [15]. However, introducing another atom, like germanium, into their structure may lead to severe disturbances of such models.

The structures of the obtained new derivatives of POSS-type compounds are analogous to those known in the literature and differ only in the presence of an atom of germanium linked via a Ge–C bond to organic groups Me, Ph, Et, vinyl or benzyl, localized at the vertex of an octahedral cage. Considering that the process of thermal decomposition is strictly related to the stability of the chemical bonds, therefore, it should be taken into account that the introduction of the germanium atom to the cage structure will reduce the stability of the structure. The bond dissociation energies of SiO_2_ and GeO_2_ have been reported to be 800 and 659 kJ/mol, respectively. The strength of the Ge–C covalent bond is 238 kJ/mol [40] and is lower than that of the Si–C bond, which is 318 kJ/mol. Another important factor that should be taken into account in the analysis of the thermal decomposition of silsesquioxane-type compounds is the effect of the intermolecular bonds [19]. However, it should be remembered that the phase transition that may occur at a temperature gradient will decrease the significance of this factor and increase that of the stability of the bonds within the molecule. In silsesquioxane-type compounds, the phase transitions take place at high temperatures (Table 2) 180–300 °C, which indicates that the intermolecular interactions are strong. In contrast to the analogous structures of an open cage [19], the benzyl derivatives containing a germanium atom at the vertex do not undergo sublimation, but at a high temperature, their phenyl groups undergo decomposition (Table 2) leaving a ceramic deposit of the composition close to that of the core of the cage structure. For each germasilisesqioxane-type compound, the glassy residue obtained after the thermal decomposition in an inert atmosphere is made of a ceramic mass containing an amorphous structure rich in Si–C bonds and containing different proportions of the Si–O and Ge–O bonds [15].

**Figure 3:**
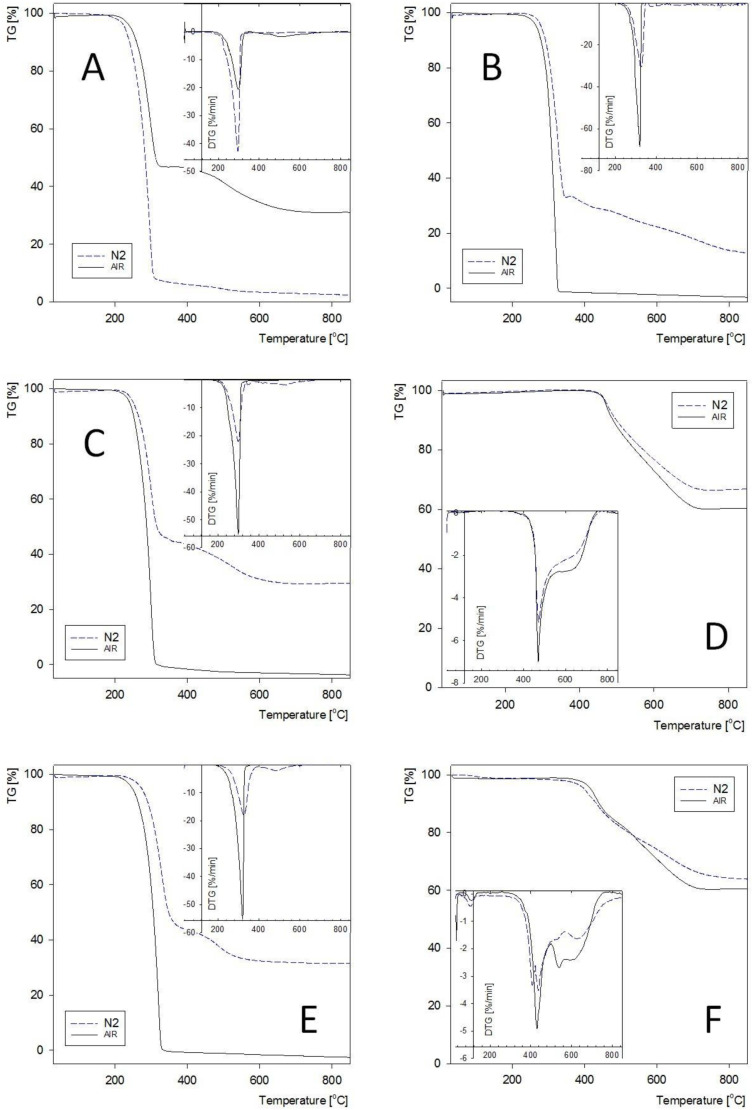

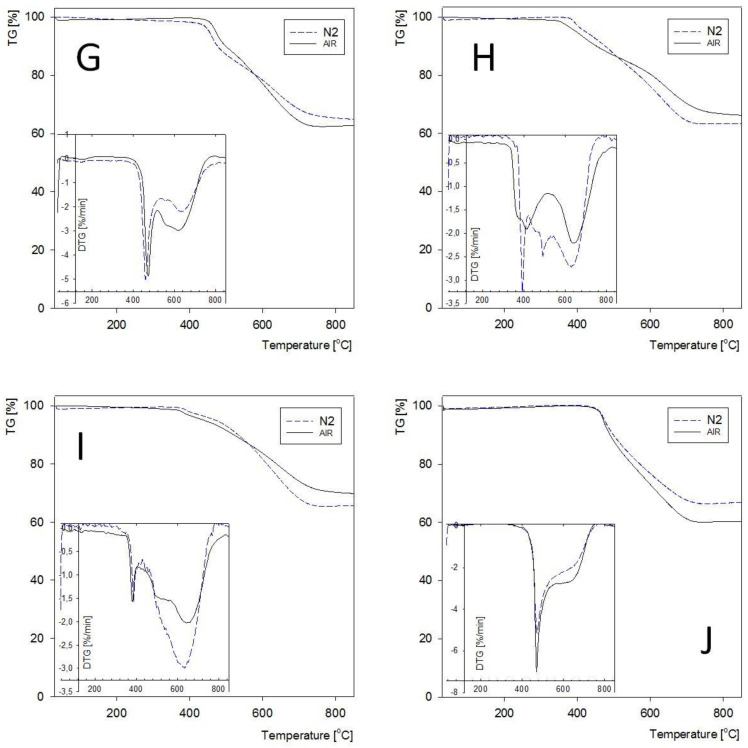
The TGA and DTG curves of obtained cubic germasilsesquioxanes. compounds No. 1–curve **A**, No. 2–curve **B**, etc. Test in oxidizing atmosphere (air)—solid line, inert atmosphere (nitrogen) dashed line.

**Figure 4:**
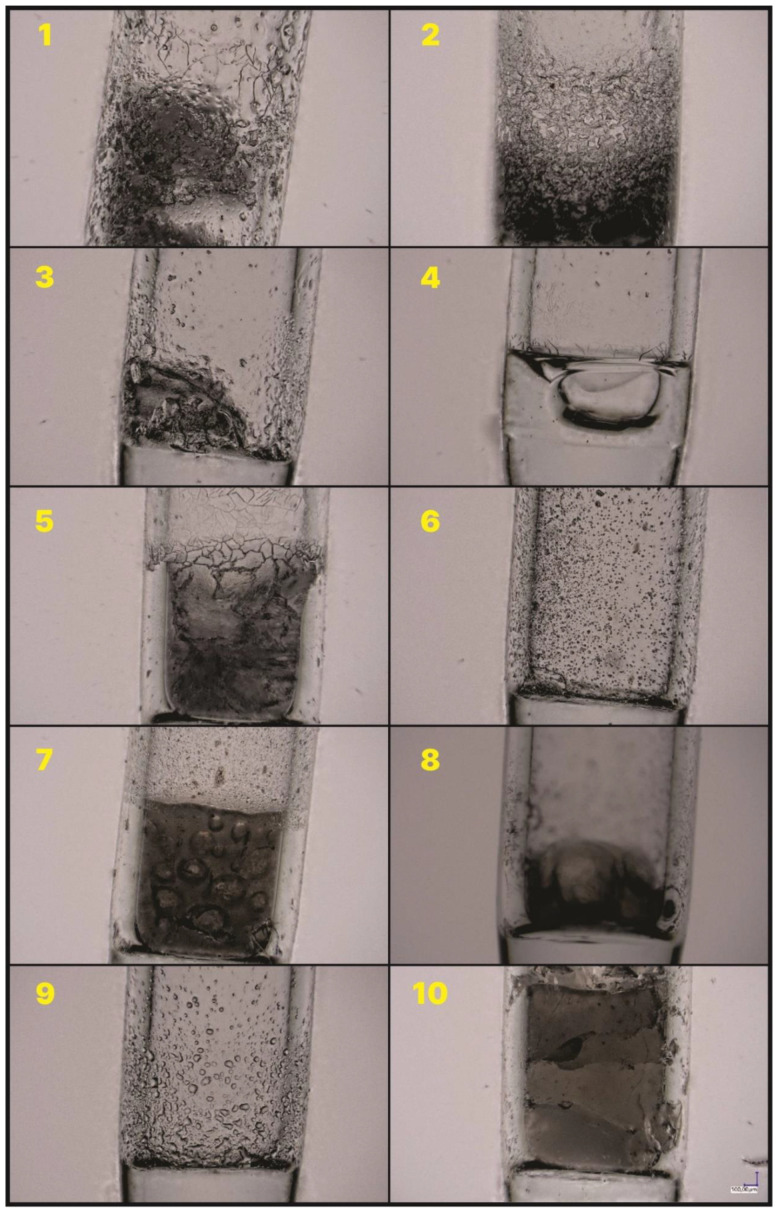
The lower part of the capillary after the heating process to 500 °C. Numbers 1–10 represent the compound given in the Table 1.

**Figure 5:**
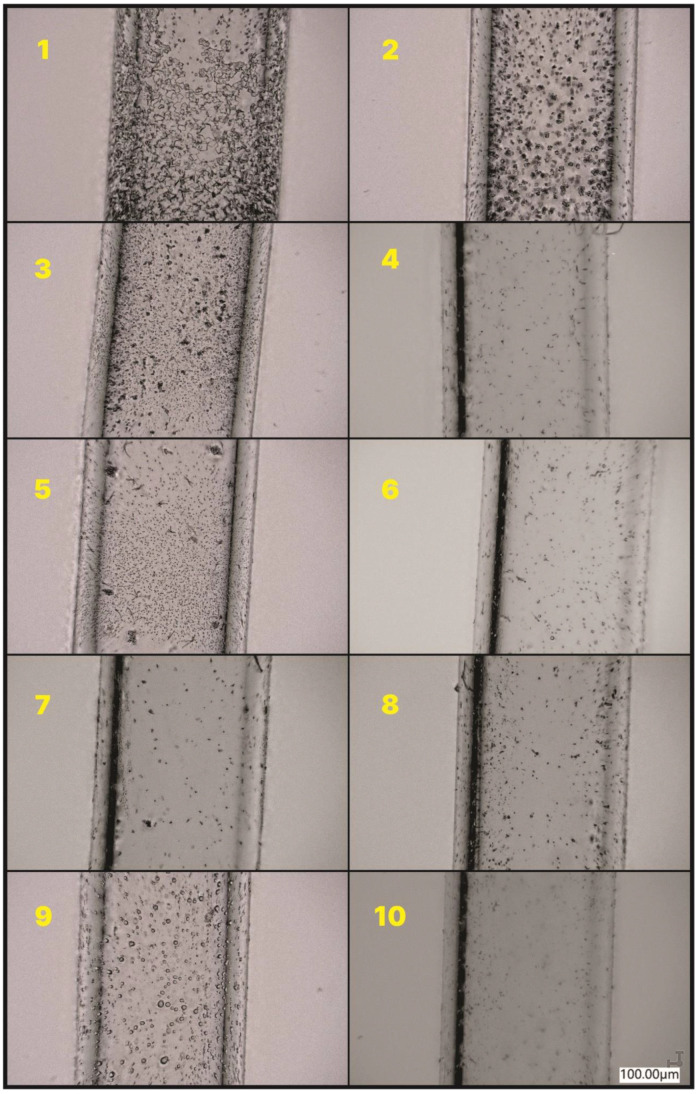
The upper part of the capillary after the heating process to 500 °C. Numbers 1–10 represent the compound given in the Table 1.

It is highly probable that the obtained ceramic material will contain a high admixture of the Ge–O and Ge–C bonds/connections. Figure 3 shows in which materials the pre-ceramic residue bring a dominant contribution. Analogously, the compounds that do not undergo pre-ceramization tend to undergo sublimation, as illustrated in Figure 4 and Figure 5. The exceptions are the vinyl group containing compounds. In contrast to their silicon analogues, they do not undergo sublimation, but the vinyl groups probably undergo polymerization with formation of polymeric structures. The dry residue obtained for the germasilsesquioxane phenyl derivatives is much greater in amount than the theoretically calculated oxide residue (for the processes taking place in either oxidizing or reducing atmosphere, see Table 2). This is a consequence of the formation of thermally stable carbon residue and stable ceramic connections Si–C and Ge–C.

**Figure 6:**
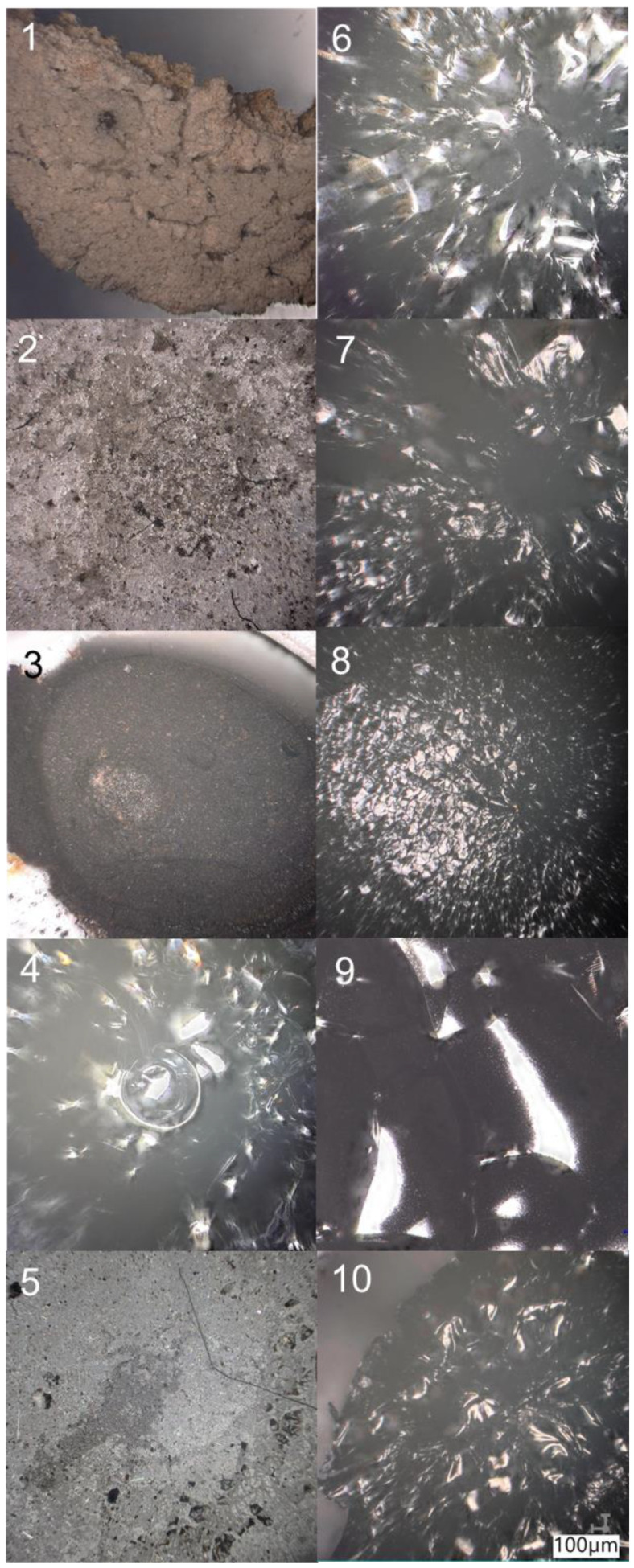
Microscopic pictures of samples and residues after heat treatment in an inert atmosphere up to 1000 °C. Numbers 1–10 represent the compound given in the Table 1.

The samples treated with the high temperature show either a glassy or porous microstructure morphology (Figure 6). In the case of the isobutyl derivatives (except for the derivative containing the vinyl group), the residue is porous (Figure 6—1–3 and 5). There is clearly a glassy morphology for the remaining materials. The next part of the research focuses on systems characterized by a glassy nature of the microstructure.

**Figure 7:**
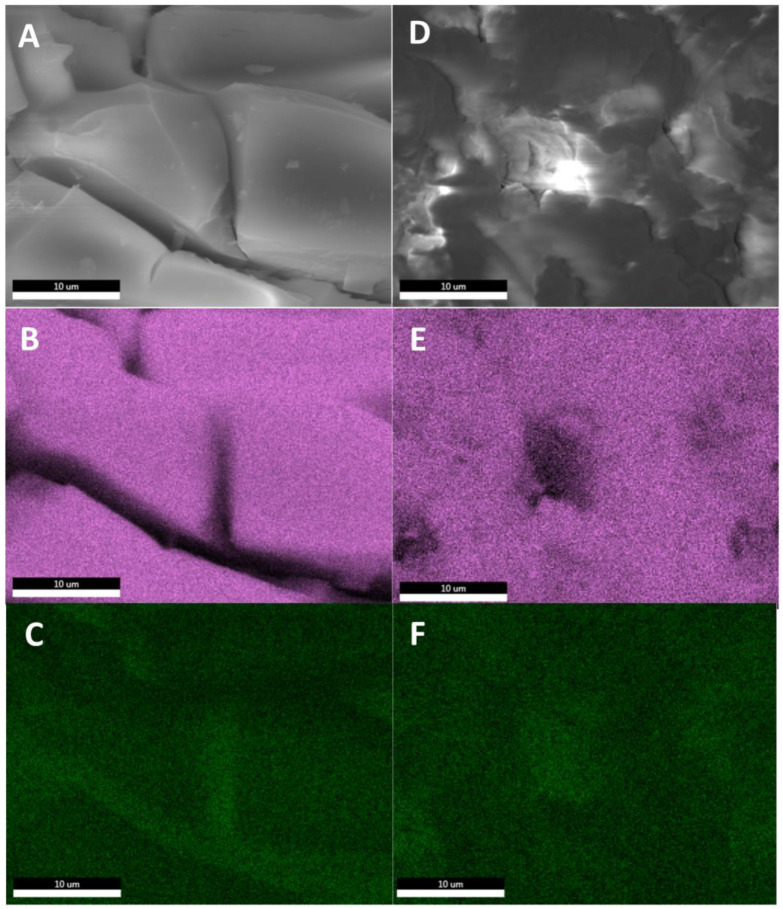
SEM-EDS images of samples **2** (**A**–**C**) and **8** (**D**–**F**) heated in an inert atmosphere up to 1000 °C. (**A**,**D**)—10,000× magnification (**B**,**E**)—silicon EDS (**C**,**F**)—germanium EDS.

Tests of the residues after thermal treatment in a more inert atmosphere showed that in the case of high-temperature treatment of the samples, the phase separation of SiO_2_ and GeO_2_ occurs (Figure 7).

**Figure 8:**
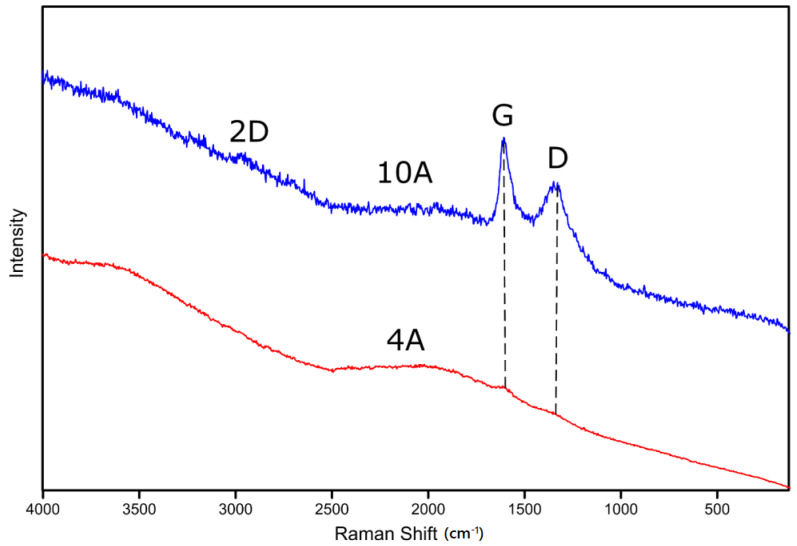
The Raman measurements of samples 4A and 10A.

The performed Raman measurements reveal that the free carbon phase is present in all tested materials. As shown in Figure 8, typical carbon bonds are present—the G and D bands [41]. The difference between the two selected spectra is the presence fluorescence. As described in the text, samples 10A–6A have Si–Ph bonds, which result in presence of well-defined bands. The G band is characteristic for the vibrations of the graphite phase in sp^2^ hybridization, while the D band, the so-called defect band, corresponds to dandling sp^3^ bonds and defects [41,42]. The presence of the 2D band indicates that the measured sample, despite a fairly weak signal and the presence of fluorescence, has probably a graphite-like phase obtained from the pyrolysis of Si-bonded functional groups, in this case phenyl groups. In the case of sample 4A, the presence of D and G bands is also visible, but their intensity is very low. In addition, it is overlapped with the presence of a very strong fluorescence signal, which probably results from the decomposition of functional groups used on the silicon atoms—iBu. The breakdown of this group generates the presence of free radical groups that contribute to this undesirable effect.

**Figure 9:**
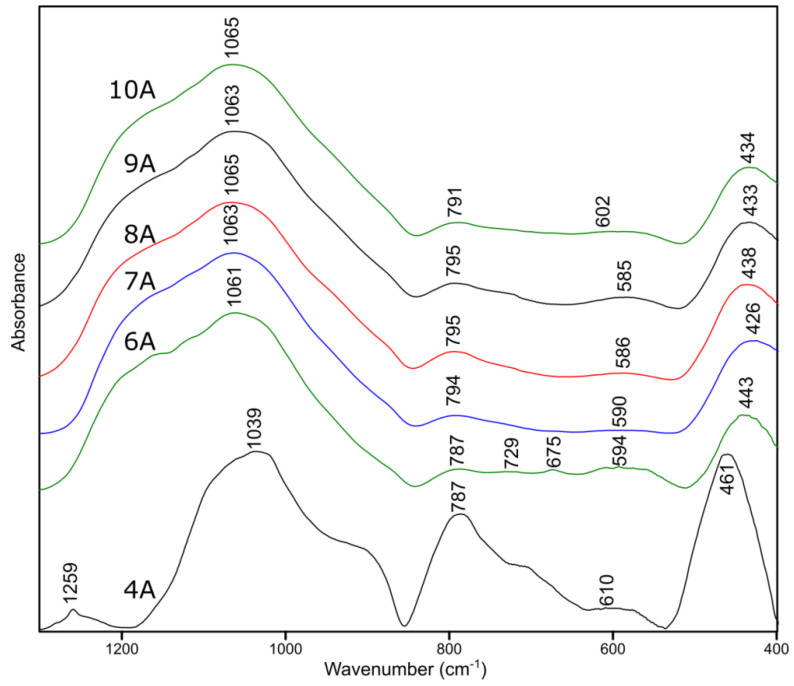
The FT-IR spectra of pyrolyzed samples.

The conducted FT–IR measurements of samples pyrolyzed in the argon atmosphere are summarized in Figure 9. As it can be seen, the spectra samples with a phenyl side group (6A−10A) are very similar.

On the other side is the 4A spectrum, which looks significantly different from the rest of the spectra in the list, as in the case of the Raman measurements. The change of the functional group at the silicon atom changes the structure of the obtained materials. The analysis of the results proves that the spectra of samples 6A–10A look like the spectra of silicon oxycarbide [43,44]. In order to analyze the structure of the studied materials in depth, the selected extreme spectra were subjected to the process of decomposition into component bands.

**Figure 10:**
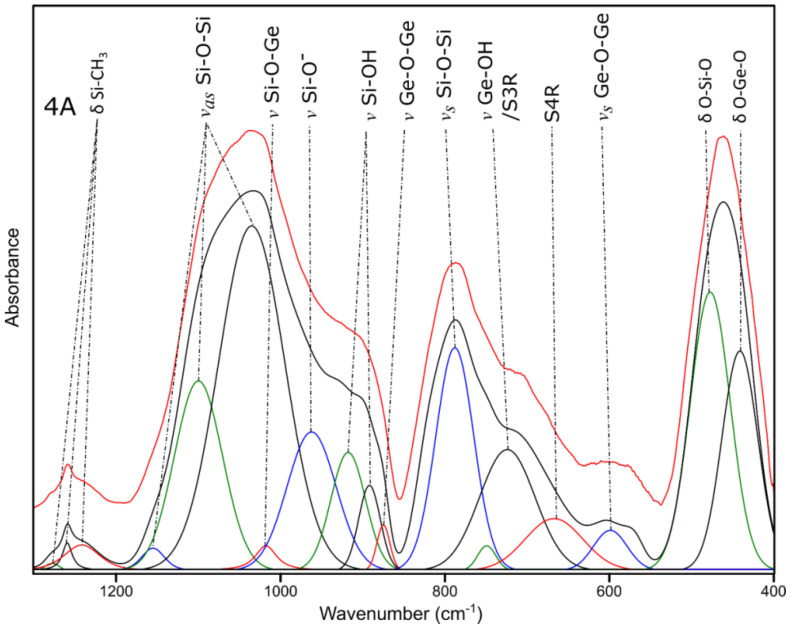
The deconvoluted 4A FT-IR spectrum.

The presented results of the decomposition for sample 4A (Figure 10) indicate the remaining organic part in the form of bands typical for Si-CH_3_ groups at approx. 1250 cm^−1^, as well as Si-O-Si vibrations in the range of 1000–1150 cm^−1^, which indicate the remains of siloxane cages [43,45]. The band at approx. 800 cm^−1^, which is characteristic for symmetrical Si-O-Si stretching vibrations, is probably also a superposition with the band characteristic for vibrations of the C-H groups [43]. At the same time, the bands from the vibrations of broken silica bridges at approx. 950 cm^−1^ are already visible, proving the ongoing process of rearrangement of bonds and the formation of the so-called black glass [43,44]. The presence of germanium is confirmed by the bands at approx. 1020, 870 and 600 cm^−1^, characteristic for the vibrations of Si-O-Ge and Ge-O-Ge [46,47].

**Figure 11:**
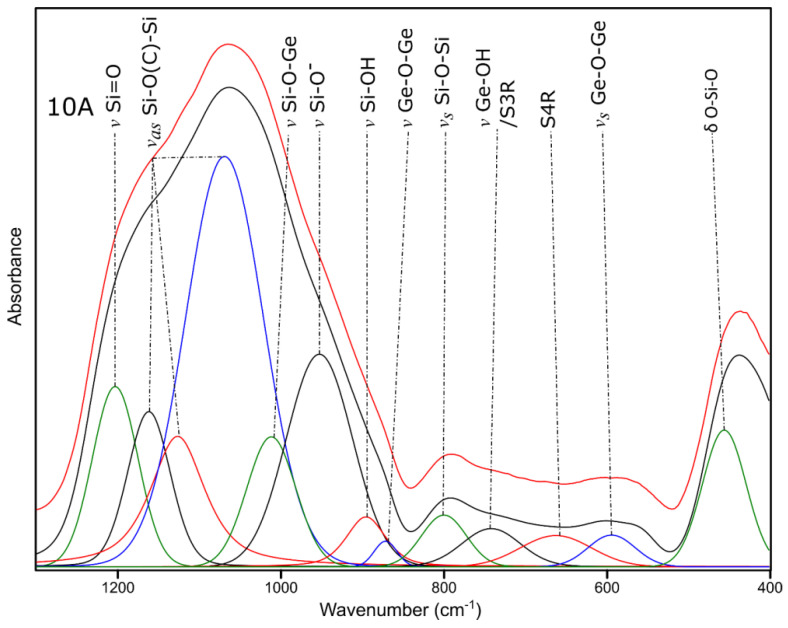
The deconvoluted 10A FT-IR spectrum.

The spectrum of the 10A sample after the decomposition process exhibits bands typical for materials from a Si-O-C system (Figure 11) [42,43]. This is confirmed by the presence of Si-O©-Si characteristic bands in the range of 1000–1200 cm^−1^ along with Si=O double bonds and Si-O^−^ [42,43]. The presence of Ge-O bonds is also confirmed, in similar wavenumbers as for sample 4A. The existence of OH species bonded to both Si and Ge [42,43,47] allows us to state that the ceramization process is not completed at the selected temperature and/or open porosity is present, which, in terms of usage of this kind of material, opens up possible applications—e.g., energy storage and catalysis.

The structural differences between the 10A and 4A samples result from the type of functional group used at the silicon atom. Its influence is visible both in Raman and FT-IR spectroscopy. In the case of the first method, the fluorescence effect in the iBut group makes it almost impossible to register any signal, while the phenyl group forms a graphite-like free carbon phase, which is confirmed by the presence of the characteristic D, G and 2D bands. In the case of FT-IR analysis, a similar relationship is observed. The use of the phenyl group leads to a structure typical for silicon oxycarbide, but this time, it is modified with Ge atoms, as evidenced by Figure 10 and Figure 11, while the second of the functional groups used causes the cage structure to be partially preserved even at a temperature of 800 °C. Structural analysis of pyrolyzed materials allows us to assume their usefulness in obtaining materials from the Si-O-C system.

## 3. Materials and Methods

### 3.1. Materials

TriSilanolIsobutyl silsesquioxane and TriSilanolPhenyl silsesquioxane were purchased from Hybrid Plastics (Hattiesburg, MI, USA). The chlorogermanes were purchased from Gelest (Morrisville, PA, USA)—benzyltrichlorogermane, ethyltrichlorogermane and phenyltrichlorogermane; and ABCR (Karlsruhe, Germany)—methyltrichlorogermane, and used as received. Vinyltrichlorogermane was prepared according to the literature’s procedure [48]. The solvents were purchased from Fisher Chemical (Mumbai, India)—THF, and Avantor Performance Materials Poland (Gliwice, Poland)—MeOH. THF was dried by distillation from benzophenone ketyl. The deuterated chloroform was purchased from Deutero (Kastellaun, Germany).

### 3.2. Synthesis of Cubic Germasilsesquioxanes ^i^Bu_7_Si_7_O_8_Ge–R

TriSilanolIsobutyl silsesquioxane (1 g, 1.26 mmol), deoxygenated and dried tetrahydrofuran (25 mL) and triethylamine (0.58 mL, 4.15 mmol) were placed in an argon-filled 50 mL single neck flask equipped with a rubber septum. The respective chlorogermanane (1.39 mmol) was then added dropwise to the reaction mixture at room temperature, which resulted in the precipitation of the ammonium salt. The suspension was vigorously stirred for 24 h at room temperature (for R = Ph and Bn, the syntheses were carried out in refluxing solution). Subsequently, the suspension was filtered through a G4 frit funnel connected to a membrane pump. The obtained filtrate was passed through a short silica gel column and then evaporated to dryness. After that, cold methanol was added to the crude powder, and the solids were collected on a G4 frit funnel. The products were obtained in the form of a white powder and thoroughly dried in vacuo on a Schlenk line.

*(Methyl)heptaisobutylgermasilsesquioxane***(1)** (Yield: 89%) ^1^H NMR (300 MHz, DCCl_3_): δ 0.6 (m, 14H), 0.72 (s, 3H, Ge-C*H*_3_), 0.95 (d, 42H), 1.86 (m, 7H); ^13^C NMR (75 MHz, DCCl_3_): δ −0.69 (Ge-*C*H_3_) 22.70, 22.79, 23.14, 24.05, 24.16, 25.88; ^29^Si NMR (79 MHz, DCCl_3_) δ −66.26, −67.65, −68.17; FT-IR: 1073.66 cm^−1^.

*(Phenyl)heptaisobutylgermasilsesquioxane***(2)** (Yield: 90%) ^1^H NMR (300 MHz, DCCl_3_): δ 0.64 (m, 14H), 0.97 (d, 42H), 1.88 (m, 7H) 7.54 (m, 5H, Ge-C_6_*H*_5_); ^13^C NMR (75 MHz, DCCl_3_): δ 22.73, 22.81, 23.15, 24.06, 24.19, 25.87, 25.91, 128.70, 130.03, 131.61, 133.30 (Ge-*C*_6_H_5_); ^29^Si NMR (79 MHz, DCCl_3_) δ −66.26, −67.65, −68.17; FT-IR: 1074.61 cm^−1^.

*(Ethyl)heptaisobutylgermasilsesquioxane***(3)** (Yield: 89%) ^1^H NMR (300 MHz, DCCl_3_): δ 0.6 (m, 14H), 0.95 (d, 42H), 1.22 (m, 5H, Ge-C*H*_2_C*H*_3_), 1.86 (m, 7H); ^13^C NMR (75 MHz, DCCl_3_): δ 6.74 (Ge-CH_2_*CH*_3_) 6.79 (Ge-C*H*_2_*C*H_3_), 22.72, 22.81, 23.17, 24.06, 24.19, 25.89; ^29^Si NMR (79 MHz, DCCl_3_) δ −66.05, −67.67, −68.21; FT-IR: 1074.94 cm^−1^.

*(Vinyl)heptaisobutylgermasilsesquioxane***(4)** (Yield: 89%) ^1^H NMR (300 MHz, DCCl_3_): δ 0.6 (m, 14H), 0.95 (m, 42H), 1.86 (m, 7H), 6.01 (dd, 1H, J_HH_ = 19.9, 12.6 Hz, CH=CH_2_), 6.11 (dd, 1H, *J_HH_* = 19.9, 3.1 Hz, CH=CH_2_), 6.19 (dd, 1H, *J_HH_* = 12.6, 3.1 Hz, CH=CH_2_);^13^C NMR (75 MHz, DCCl_3_): δ 22.60, 22.92, 23.86, 23.97, 25.68, 25.72, 126.50 (=*C*H_2_), 137.12 (=CH−Ge); ^29^Si NMR (79 MHz, DCCl_3_) δ −66.26, −67.65, −68.17; MS (ESI); FT-IR: 1075.14 cm^−1^.

*(Benzyl)heptaisobutylgermasilsesquioxane***(5)** (Yield: 88%) ^1^H NMR (300 MHz, DCCl_3_): δ 0.59 (m, 14H), 0.95 (d, 42H), 1.83 (m, 7H), 2.72 (s, 2H, Ge-CH_2_-Ph), 7.24 (m, 5H, Ge-CH_2_-C_6_H_5_); ^13^C NMR (75 MHz, DCCl_3_): δ 22.61, 22.94, 23.88, 25.72, 24.81 (Ge-*C*H_2_-Ph), 125.80, 128.39, 128.98, 133.68 (Ge-CH_2_-*C*_6_H_5_); ^29^Si NMR (79 MHz, DCCl_3_) δ −65.82, −67.67, −68.24; FT-IR: 1073.93 cm^−1^.

### 3.3. Synthesis of Cubic Germasilsesquioxanes Ph_7_Si_7_O_8_Ge–R

Similarly, the syntheses were carried out as described in Section 3.2, using TriSilanolPhenyl POSS (1 g, 1.07 mmol) as a starting material.

*(Methyl)heptaphenylgermasilsesquioxane***(6)** (Yield: 90%) ^1^H NMR (300 MHz, DCCl_3_): δ 0.89 (s, 3H, Ge-CH_3_), 7.42 (m, 35H); ^13^C NMR (75 MHz, DCCl_3_)**:** δ −0.42 (Ge-*C*H_3_) 127.98, 130.73, 130.81, 131.59, 134.38; ^29^Si NMR (79 MHz, DCCl_3_) δ −77.45, −78.11, −78.57; FT-IR: 1058.85 cm^−^^1^.

*Octaphenylgermasilsesquioxane***(7)** (Yield: 42%) ^1^H NMR (300 MHz, DCCl_3_): δ ^13^C NMR (75 MHz, DCCl_3_)**:** δ 127.69, 130.54 133.15, 134.14 ^29^Si NMR (79 MHz, DCCl_3_) δ −77.48, −78.16, −78.61; FT-IR: 1057.95 cm^−1^.

*(Ethyl)heptaphenylgermasilsesquioxane***(8)** (Yield: 88%) ^1^H NMR (300 MHz, DCCl_3_): δ ^13^C NMR (75 MHz, CDCl_3_): δ 6.86 (Ge-CH_2_*C*H_3_), 10.05 (Ge-*C*H_2_CH_3_), 127.85, 130.54, 131.56, 134.11, 134.25; ^29^Si NMR (79 MHz, DCCl_3_) δ −77.22, −78.12, −78.59; FT-IR: 1059.09 cm^−1^.

*(Vinyl)heptaphenylgermasilsesquioxane***(9)** (Yield: 90%) ^1^H NMR (300 MHz, DCCl_3_): δ 6.16 (dd, 1H, *J_HH_* = 19.8, 12.3 Hz, CH=CH_2_), 6.25 (dd, 1H, *J_HH_* = 17.1, 2.7 Hz, CH=CH_2_), 6.30 (dd, 1H, *J_HH_* = 9.7, 2.7 Hz, CH=CH_2_), 7.56 (m, 35H); ^13^C NMR (75 MHz, DCCl_3_)**:** δ *127.78* (=*C*H_2_), 130.44, 130.52, 130.63, 131.19, 134.17 (=CH−Ge); ^29^Si NMR (79 MHz, DCCl_3_) δ −76.96, −78.12, −78.58; FT-IR: 1057.95 cm^−1^.

*(Benzyl)heptaphenylgermasilsesquioxane***(10)** (Yield: 87%) ^1^H NMR (300 MHz, DCCl_3_): δ 2.86 (s, 2H, Ge-CH_2_-Ph), 7.38 (m, 40H); ^13^C NMR (75 MHz, DCCl_3_)**:** δ 25.08 (Ge-*C*H_2_-Ph), 126.38, 127.87, 127.92, 128.87, 129.24, 130.63, 130.76, 131.34, 132.96, 134,92, 134.35; ^29^Si NMR (79 MHz, DCCl_3_) δ −77.22, −78.12, −78.59; FT-IR: 1058.41 cm^−1^.

### 3.4. Analytical Methods

The ^1^H NMR spectra were recorded on a Bruker Ultrashield 300 MHz (Billerica, MA, USA). The ^13^C and ^29^Si NMR spectra were recorded on a Bruker Ascend (Billerica, MA, USA) 400 MHz operating at 101 and 79 MHz, respectively. Benzene-*d_6_* was used as a solvent.

The MALDI-TOF mass spectra were recorded on an UltrafleXtreme mass spectrometer by Bruker Daltonics (Billerica, MA, USA) equipped with a SmartBeam II laser (355 nm) in the 500–4000 *m*/*z* range, and 2,5-dihydroxybenzoic acid (DHB) served as the matrix.

Thermogravimetry (TG) was performed using a NETZSCH 209 F1 Libra gravimetric analyzer (Selb, Germany). Samples of 2 ± 0.2 mg were placed in Al_2_O_3_ crucibles. Measurements were carried out under nitrogen (flow of 20 mL/min) in the range of 50–800 °C and at a 20 °C/min heating rate.

Melting point was measured using a Büchi M-565 analyzer (Flawil, Switzerland). Based on the optical parameters of the phase change course of the sample in the capillary, an automatic measurement was performed with the determination of the melting point (temperature increase of 5 °C/min). The result is the average of the three measurements.

The capillaries’ photos were analyzed under a Digital Light Microscope Keyence VHX 7000 with 100× to 1000× VH-Z100T lens (Osaka, Japan). All of the pictures were recorded with a VHX 7020 camera.

The energy-dispersive spectroscopy (EDS) analyses were conducted at a beam acceleration voltage of 15 kV using an EDAX Octane SDD detector. The EDS maps of element overlay were made at a resolution of 0.3 µm.

The FTIR measurements were carried out using a Thermo Scientific Nicolet iS50 FTIR spectrometer (Mumbai, India). A total of 2 mg of samples were mixed with 200 mg of KBr and pressed at 10 tons press into the form of discs.

The FT-IR measurements were performer with the help of a Bruker Vertex 70v spectrometer (Billerica, MA, USA). The standard KBr pellet method was used. A total of 256 scans in the range of 4000–400 cm^−1^ were collected with 2 cm^−1^ resolution. The obtained spectra were subjected to baseline correction and normalization using Bruker OPUS 7.2 software (Billerica, MA, USA).

The fitting procedure was carried out using Bruker OPUS 7.2 software. A Levenberg-Marquart algorithm was used. The RMS noise after deconvolution process was below 1.

The Raman studies were carried out using a WITec Alpha 300M+ spectrometer (Ulm, Germany). A 488 nm laser with 600 grating was chosen along with a 100× ZEISS objective (Oberkochen, Germany). Each sample was measured 3 times for 2 min each.

## 4. Conclusions

The introduction of a germanium atom to the cage structure of POSS type weakens the stability of the structure relative to that of the analogous one based on silicon. The vinyl derivatives have a tendency towards cross-linking, dimerization or polymerization, leading to decomposition with a low emission of volatiles. Their behavior can be used for the production of pre-ceramic systems, in particular the Si–C materials admixtured with Ge–C domains.

The POSS derivatives with phenyl substituents do not show a tendency towards sublimation; with increasing temperatures, they undergo decomposition, emitting volatile organic residues and leaving a large amount of solid residue. Therefore, these derivatives can be used as pre-ceramic materials. The isobutyl derivatives with Me, Ph, Bn or Et substituents undergo effective sublimation that is undisturbed with chemical reactions, which can be used for the purification of a given derivative from the post-reaction mixture. In the processes of thermal decomposition, some parts of silicon and germanium atoms become components of volatile species and are liberated from the dry residue.

## Figures and Tables

**Table 1:** The cubic germasilsesquioxanes obtained in this study.

^i^Bu_7_Si_7_O_8_Ge–R		Ph_7_Si_7_O_8_Ge–R	
No.	R	No.	R
**1**	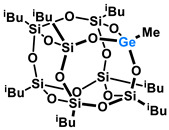	**6**	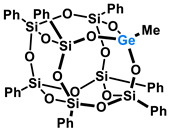
**2**	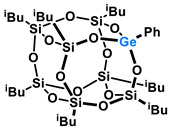	**7**	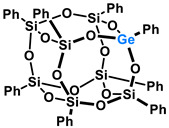
**3**	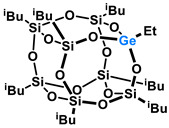	**8**	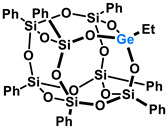
**4**	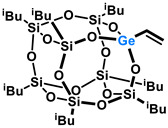	**9**	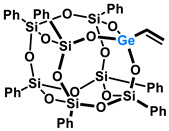
**5**	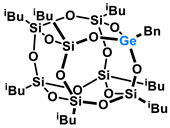	**10**	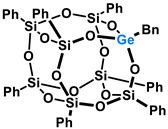

**Table 2:** A summary of the TG data from the measurements.

						Air				Nitrogen			
Ge POSS	M	m_C_ [%]	m_A_ [%]	m_B_ [%]	p_D_ [%]	p_TG_ [%]	P_5_ [%]	P_98_ [%]	DTG_max_ [°C]	p_TG_ [%]	P_5_ [%]	P_98_ [%]	DTG_max_ [°C]
**1**	876	53.5	38.79	1.72	59.9%	46.7	246	751	297	2.6	229	752	304
**2**	938	50.0	36.23	8.22	55.9%	47.3	278	756	320	0	267	329	320
**3**	890	52.7	38.18	3.26	58.9%	44.5	252	696	300	0	242	314	300
**4**	888	52.8	38.27	3.05	59.1%	56.24	303	660	N.a.	13.5	238	770	Na.
**5**	952	49.3	35.69	9.57	55.1%	31.5	276	680	329	0	250	336	321
**6**	1016	46.2	53.13	1.48	51.6%	60.3	424	741	431	63.8	410	>850	407/437
**7**	1078	43.5	50.07	7.15	48.7%	58.8	472	755	467	52.6	460	719	466
**8**	1030	45.6	52.4	2.82	50.9%	63.3	422	738	394/631	66.1	397	828	412/639
**9**	1028	45.6	52.5	2.63	51.0%	65.4	459	773	378/634	69.9	467	>850	382/646
**10**	1092	43.0	49.43	8.34	48.0%	66.4	475	753	472	60.0	471	740	470

**M**—molar mass, **m_C_**—core mass, **m_A_**—mass of A groups, **m_B_**—mass of B groups, **p_D_**—theoretical dry residue, **p_TG_**—TG dry residue, **p_5_**—5% of the efect, **p_98_**—98% max. of the efect, **DTG_max_**—1st DTG max.

## Sample Availability

Not available.

## References

[1] Hartmann-Thompson C. (2011). Applications of Polyhedral Oligomeric Silsesquioxanes.

[2] Soldatov M., Liu H. (2021). Hybrid porous polymers based on cage-like organosiloxanes: Synthesis, properties and applications. Prog. Polym. Sci..

[3] Lickiss P.D., Cordess D.B., Hill A.F., Fink M.J. (2008). Fully Condensed Polyhedral Oligosilsesquioxanes (POSS): From Synthesis to Application. Advances in Organometallic Chemistry.

[4] Dirè S., Borovin E., Ribot F., Klein L., Aparicio M., Jitianu A. (2018). Architecture of Silsesquioxanes. Handbook of Sol-Gel Science and Technology.

[5] Matisons J., Marciniec B., Maciejewski H., Pietraszuk C. (2011). Applications of Polyhedral Oligomeric Silsesquioxanes. Advances in Silicon Science.

[6] Frydrych M., Pakuła D., Sztorch B., Brząkalski D., Przekop R.E., Marciniec B. (2021). Novel Silsesquioxane-Derived Boronate Esters—Synthesis and Thermal Properties. Molecules.

[7] Kalia S., Pielichowski K. (2018). Polymer/POSS Nanocomposites and Hybrid Materials: Preparation, Properties, Applications.

[8] Qian Y., Wei P., Zhao X., Jiang P., Yu H. (2013). Flame retardancy and thermal stability of polyhedral oligomeric silsesquioxane nanocomposites. Fire Mater..

[9] Cordes D.B., Lickiss P.D., Rataboul F. (2010). Recent Developments in the Chemistry of Cubic Polyhedral Oligosilsesquioxanes. Chem. Rev..

[10] Lorenz V., Edelman F.T., West R., Hill A. (2005). Metallasilsesquioxanes. Advances in Organometallic Chemistry.

[11] Nanjo M., Sasage T., Mochida K. (2003). Synthesis and characterization of alkylgermasesquioxanes. J. Organomet. Chem..

[12] Duverneuil G., Mazerolles P., Perrier E. (1995). Polygermoxanes suitable for biochemical purposes: II Linear trigermoxanes (high-viscosity oils). Appl. Organometal. Chem..

[13] Risen W.M., Wang Y.Z., Honore A. (2001). Germanosiloxane Materials and Optical Components Comprising the Same. U.S. Patent.

[14] Fina A., Tabuani D., Carniato F., Frache A., Boccaleri E., Camino G. (2006). Polyhedral oligomeric silsesquioxanes (POSS) thermal degradation. Thermochim. Acta.

[15] Ghani K., Keshavarz M.H., Jafari M., Khademian F. (2018). A novel method for predicting decomposition onset temperature of cubic polyhedral oligomeric silsesquioxane derivatives. J. Therm. Anal. Calorim..

[16] Moore M., Ramirez S.M., Yandek G.R., Haddad T.S., Mabry J.M. (2011). Asymmetric aryl polyhedral oligomeric silsesquioxanes (ArPOSS) with enhanced solubility. J. Organomet. Chem..

[17] Blanco I., Bottino F.A., Abate L. (2016). Influence of n-alkyl substituents on the thermal behaviour of Polyhedral Oligomeric Silsesquioxanes (POSSs) with different cage’s periphery. Thermochim. Acta.

[18] Endo H., Takeda N., Unno M. (2014). Synthesis and Properties of Phenylsilsesquioxanes with Ladder and Double-Decker Structures. Organometallics.

[19] Yuasa S., Sato Y., Imoto H., Naka K. (2019). Thermal Properties of Open-Cage Silsesquioxanes: The Effect of Substituents at the Corners and Opening Moieties. Bull. Chem. Soc. Jpn..

[20] Tanaka K., Chujo Y. (2012). Advanced functional materials based on polyhedral oligomeric silsesquioxane (POSS). J. Mater. Chem..

[21] Li G., Wang L., Ni H., Pittman C.U. (2001). Polyhedral Oligomeric Silsesquioxane (POSS) Polymers and Copolymers: A Review. J. Inorg. Organomet. Polym. Mater..

[22] Blanco I. (2018). The Rediscovery of POSS: A Molecule Rather than a Filler. Polymers.

[23] Tanaka K., Chujo Y. (2013). Unique properties of amphiphilic POSS and their applications. Polym. J..

[24] Dua Y., Liu H. (2020). Cage-like silsesquioxanes-based hybrid materials. Dalton Trans..

[25] Wang J., Du W., Zhang Z., Gao W., Li Z.J. (2021). Biomass/polyhedral oligomeric silsesquioxane nanocomposites: Advances in preparation strategies and performances. Appl. Polym. Sci..

[26] O’Lenick A. (2008). Silicones for Personal Care.

[27] Frąckowiak D., Żak P., Spólnik G., Pyziak M., Marciniec B. (2015). New Vinylgermanium Derivatives of Silsesquioxanes and Their Ruthenium Complexes—Synthesis, Structure, and Reactivity. Organometallics.

[28] Żak P., Frąckowiak D., Grzelak M., Bołt M., Kubicki M., Marciniec B. (2016). Olefin Metathesis of Vinylgermanium Derivatives as Method for the Synthesis of Functionalized Cubic and Double-Decker Germasilsesquioxanes. Adv. Synth. Catal..

[29] Kaźmierczak J., Hreczycho G. (2017). Catalytic Approach to Germanium-Functionalized Silsesquioxanes and Germasilsesquioxanes. Organometallics.

[30] Brząkalski D., Przekop R.E., Dobrosielska M., Sztorch B., Marciniak P., Marciniec B. (2020). Highly bulky spherosilicates as functional additives for polyethylene processing—Influence on mechanical and thermal properties. Polym. Compos..

[31] Brząkalski D., Przekop R.E., Sztorch B., Jakubowska P., Jałbrzykowski M., Marciniec B. (2020). Silsesquioxane Derivatives as Functional Additives for Preparation of Polyethylene-Based Composites: A Case of Trisilanol Melt-Condensation. Polymers.

[32] Brząkalski D., Sztorch B., Frydrych M., Pakuła D., Dydek K., Kozera R., Boczkowska A., Marciniec B., Przekop R.E. (2020). Limonene Derivative of Spherosilicate as a Polylactide Modifier for Applications in 3D Printing Technology. Molecules.

[33] Brząkalski D., Przekop R.E., Sztorch B., Frydrych M., Pakuła D., Jałbrzykowski M., Markiewicz G., Marciniec B. (2021). Why POSS-Type Compounds Should Be Considered Nanomodifiers, Not Nanofillers—A Polypropylene Blends Case Study. Polymers.

[34] Niemczyk A., Dziubek K., Sacher-Majewska B., Czaja K., Czech-Polak J., Oliwa R., Lenża J., Szołyga M. (2018). Thermal Stability and Flame Retardancy of Polypropylene Composites Containing Siloxane-Silsesquioxane Resins. Polymers.

[35] Liu H., Zheng S. (2005). Polyurethane Networks Nanoreinforced by Polyhedral Oligomeric Silsesquioxane. Macromol. Rapid Commun..

[36] Blanco I., Bottino F.A., Bottino P. (2012). Influence of symmetry/asymmetry of the nanoparticles structure on the thermal stability of polyhedral oligomeric silsesquioxane/polystyrene nanocomposites. Polym. Compos..

[37] Mohamed M.G., Kuo S.W. (2019). Functional Polyimide/Polyhedral Oligomeric Silsesquioxane Nanocomposites. Polymers.

[38] Bik M., Gil A., Stygar M., Dąbrowa J., Jeleń P., Długoń E., Leśniak M., Sitarz M. (2019). Studies on the oxidation resistance of SiOC glasses coated TiAl alloy. Intermetallics.

[39] Bik M., Stygar M., Jeleń P., Dąbrowa J., Leśniak M., Brylewski T., Sitarz M. (2017). Protective-conducting coatings based on black glasses (SiOC) for application in Solid Oxide Fuel Cells. Int. J. Hydrogen Energy.

[40] Lide D.R. (2005). CRC Handbook of Physics and Chemistry.

[41] Ferrari A.C., Robertson J. (2000). Interpretation of Raman spectra of disordered and amorphous carbon. Phys. Rev. B.

[42] Jeleń P., Bik M., Nocuń M., Gawęda M., Długoń E., Sitarz M. (2016). Free carbon phase in SiOC glasses derived from ladder-like silsesquioxanes. J. Mol. Struct..

[43] Bik M., Jeleń P., Długoń E., Bik E., Mroczka K., Barańska M., Sitarz M. (2019). SiAlOC glasses derived from sol-gel synthesized ladder-like silsesquioxanes. Ceram. Int..

[44] Jeleń P., Szumera M., Gawęda M., Długoń E., Sitarz M. (2017). Thermal evolution of ladder-like silsesquioxanes during formation of black glasses. J. Therm. Anal. Calorim..

[45] Wang B., Shi M., Ding J., Huang Z. (2021). Polyhedral oligomeric silsesquioxane (POSS)-modified phenolic resin: Synthesis and anti-oxidation properties. e-Polymers.

[46] Arnold D.C., Hobbs R.G., Zirngast M., Marschner C., Hill J.J., Ziegler K.J., Morris M.A., Holmes J.D. (2009). Single step synthesis of Ge–SiO x core-shell heterostructured nanowires. J. Mater. Chem..

[47] Groza A., Surmeian A. (2015). Characterization of the oxides present in a polydimethylsiloxane layer obtained by polymerisation of its liquid precursor in corona discharge. J. Nanomater..

[48] Faller J.W., Kultyshev R.G. (2002). Palladium-Catalyzed Cross-Coupling Reactions of Allyl, Phenyl, Alkenyl, and Alkynyl Germatranes with Aryl Iodides. Organometallics.

